# FOXM1/LINC00152 feedback loop regulates proliferation and apoptosis in rheumatoid arthritis fibroblast-like synoviocytes via Wnt/β-catenin signaling pathway

**DOI:** 10.1042/BSR20191900

**Published:** 2020-01-21

**Authors:** Wenlong Wang, Piaopiao Guo, Mengjie Chen, Die Chen, Yongjun Cheng, Long He

**Affiliations:** 1 Department of Rheumatology, the First People’s Hospital of Wenling, Wenling, 317500 Zhejiang, China; 2 Department of laboratory, the First People’s Hospital of Wenling, Wenling, 317500 Zhejiang, China

**Keywords:** FOXM1, LINC00152, RA FLS, Wnt/β-catenin signaling pathway

## Abstract

Rheumatoid arthritis (RA), a chronic systemic disease, is featured with inflammatory synovitis, which can lead to destruction on bone and cartilage and even cause disability. Emerging studies demonstrated that Fibroblast-like synoviocytes (FLS) is a vital cellular participant in RA progression. Long non-coding RNAs (lncRNAs) are also reported to participate in the pathogenesis of RA. In our present study, lncRNA microarray analysis was applied to screen out lncRNAs differentially expressed in RA FLS. Among which, cytoskeleton regulator RNA (LINC00152) presented biggest fold change. Gain- or loss-of function assays were further carried out in RA FLS, and the results revealed that LINC00152 promoted proliferation but induced apoptosis in RA FLS. Furthermore, up-regulation of LINC00152 may induce promotion of Wnt/β-catenin signaling pathway in RA FLS. Mechanistically, we found that forkhead box M1 (FOXM1) transcriptionally activated LINC00152 in RA FLS. Additionally, LINC00152 positively regulated FOXM1 via sponging miR-1270. In conclusion, the present study focused on elucidating the function of FOXM1/LINC00152 positive feedback loop in RA FLS and its association with Wnt/β-catenin signaling.

## Introduction

Rheumatoid arthritis (RA), a chronic systemic disease, is featured with inflammatory synovitis, which can lead to destruction on bone and cartilage and even cause disability [1,2]. The incidence of RA is higher in women and older people than that in man and younger people [3]. The pathogenesis of RA is induced by complicated factors, including alteration of gene expression, environmental change and other reasons [4]. Outcomes of RA patients experiencing longer time will be worse. Thus, diagnosis of RA patients in early stage is essential for effective treatment [5]. Hence, deep understanding of the molecular mechanism involved in RA development is necessary to find novel efficient diagnostic method.

Long non-coding RNAs (lncRNAs), a class of transcripts with restrained protein-coding ability, are crucial regulators in numerous biological processes, such as the proliferation and apoptosis [6,7]. Recently, lncRNAs are acknowledged as participants in the pathogenesis of autoimmune diseases, including RA [8,9]. Fibroblast-like synoviocytes (FLS), behaving like fibroblasts, are able to generate proteins to lubricate the joint. Emerging papers demonstrated that FLS is a crucial participant in RA [10–12]. Differential expression of lncRNAs can also affect proliferation, apoptosis and migration of FLS derived from RA patients [13–15]. Thus, investigating novel functional lncRNAs in RA FLS cellular processes is important for finding novel diagnostic biomarkers for RA patients.

In our present study, lncRNA microarray analysis was applied to screen out differentially expressed in RA FLS. Among which, cytoskeleton regulator RNA (LINC00152) presented biggest fold change. Gain- or loss-of function assays were further implemented in RA FLS to assess the role of LINC00152 in regulating proliferation and apoptosis of RA FLS. Wnt/β-catenin signaling pathway has been reported as a participant in RA progression [16,17]. Here, we explored the correlation between LINC00152 and Wnt/β-catenin signaling in RA FLS. Mechanistically, we investigated the regulatory role of forkhead box M1 (FOXM1) on LINC00152 transcription in RA FLS. Additionally, regulatory effect of LINC00152 on FOXM1 expression was also detected. In summary, the present study focused on elucidating the function of FOXM1/LINC00152 positive feedback loop in RA FLS and its association with Wnt/β-catenin pathway.

## Materials and methods

### Bioinformatics analysis

FOXM1 is predicted as the transcription regulator for LINC00152 in accordance with the ChIP-seq result of UCSC (http://genome.ucsc.edu/). Binding sequence between miR-194-5p and LINC00152 was predicted from DIANA (http://carolina.imis.athena-innovation.gr/diana_tools/web/index.php?r=lncbasev2%2Findex-predicted). Putative binding sites of miR-194-5p in 3′UTR of FOXM1 were obtained from starbase (http://starbase.sysu.edu.cn/).

### Microarray analysis

TRIzol reagent from Thermo Fisher Scientific (Waltham, MA, U.S.A.) was utilized in order to harvest total RNAs. Label and hybridization of synthetic cDNA with lncRNA microarray chips were achieved by Cyanine-3-CTP-labeled cRNA. The various expressions of lncRNAs were sorted out via whole-genome microarray expression profiling. The lncRNAs microarray analysis was accordingly performed, and R program was applied for clustering differentially expressed genes.

### Cell culture

Normal human FLSs cells and RA FLSs cells were acquired from the Shanghai Cell Bank of the Chinese Academy of Sciences (Shanghai, China). Cells were grown in DMEM (Invitrogen, Carlsbad, CA, U.S.A.) with additional 10% fetal bovine serum (FBS; HyClone, UT, U.S.A.) plus 1% antibiotics (penicillin/streptomycin; Invitrogen) at 37°C under humidified atmosphere with 5% CO_2_. Medium was changed every fourth day.

### Cell transfection

RA FLSs were innoculated into 6-well plates and thereafter incubated. The shRNAs against LINC00152 (sh-LINC00152#1 and sh-LINC00152#2) and FOXM1 (sh-FOXM1#1 and sh-FOXM1#2) and their negative controls, along with miR-1270 mimics and its NC, were all bought from Genepharma (Shanghai, China). Transfection was carried out with the use of Lipotransfectamine 3000 (Thermo Fisher Scientific) for 48 h.

### Quantitative real-time PCR

For determining the relative expression, total RNA was extracted from RA FLSs cells. The Verso Reverse transcription kit (Thermo Fisher Scientic) was applied to synthesize cDNA based on the supplier’s standard. Relative expression was quantified via SYBR Green PCR kit (Thermo Fisher Scientific) on the ABI 7300 (Applied Biosystems, Foster City, CA, U.S.A.). Here, GAPDH and U6 were used as housekeeping genes. Results were measured using the 2^−ΔΔ*C*^_t_ method.

### TUNEL assay

Evaluation of cell death was carried out using TUNEL assay with the help of an *in situ* cell death detection kit (Hoffman-La Roche, Basel, Switzerland) as per instructions of the supplier. Five fields in equal size were chosen at random and analyzed using a microscope (Olympus, Tokyo, Japan). Cell nuclei were in the blue regions, and apoptotic cells were in the green regions.

### CCK-8 assay

The proliferation of transfected RA FLS was assessed utilizing the CCK-8 detection kit (Beyotime, Shanghai, China) as the manufacturer requested. Cells (5 × 10^4^ cells/ml) were incubated in 96-well plates for 24 h. After that, cells were treated with resveratrol (Adooq Bioscience, Nanjing, China). Subsequently, CCK-8 solution (20 μl/well) was added and cells were co-incubated for 2 h at 37°C. At last, the absorbance was measured at 450 nm.

### Flow cytometry analysis

For cell-cycle analysis, RA FLS (5 × 10^4^ cells) following LINC00152 deficiency were harvested, accompanied with PBS washing. propidium iodide (PI) staining solution (RNase A 100 μg/ml and PI 500 μg/ml) was adopted for the incubation of RA FLS at 4°C for 30 min. Flow cytometry was used for analysis.

### EdU assay

Transfected RA FLSs cells were put into 96-well plates. After adding EdU (10 µmol/l; RiboBio, Guangdong, China), cells were cultivated for 2 h. Cells were then fixed in phosphate-buffered saline (PBS; Thermo Fisher Scientific) with paraformaldehyde (PFA; Sigma-Aldrich, St. Louis, U.S.A.) and washed in PBS twice with use of bovine serum albumin (BSA; Sigma-Aldrich). To permeabilize cells, PBS with 0.5% Triton X-100 (Solarbio, Beijing, China) was used. Cells were then stained with DAPI (Sigma–Aldrich) and rinsed by PBS. The EdU positive cells were observed applying a fluorescence microscope (Pudan optical instrument, Shanghai, China).

### Western blotting

Total protein was extracted by lysing transfected RA FLSs in RIPA buffer (Sigma-Aldrich) and measured with BCA Protein Assay kit (Beyotime) based on the protocol provided by supplier. Cell lysates were then isolated on 10% sodium dodecyl sulfate polyacrylamide gel electrophoresis (SDS-PAGE; Bio-Rad Laboratories, Hercules, CA, U.S.A.), followed by transferring to polyvinylidene fluoride (PVDF) membranes (Millipore, Billerica, MA, U.S.A.). Subsequently, the PVDF membranes were blocked utilizing 5% skimmed milk for around 1 h at room temperature, and sequentially incubated with primary antibodies against β-catenin (1/7000, ab32572, Abcam, Cambridge, U.S.A.), Cyclin D1 (1/100, ab16663, Abcam), C-myc (1/1000, ab32072, Abcam), FOXM1 (1/1000, ab180710, Abcam) and GAPDH (1/1000, ab8245, Abcam) and then with secondary antibodies. ECL Western blot kit (Thermo Fisher Scientific) was employed to observe protein bands.

### RNA isolation of nuclear and cytoplasmic fractions

After RA FLSs were reaped, they were maintained in cell fractionation buffer and then centrifuged. After that, the cell supernatant was harvested as cytoplasmic fraction, while the remaining lysates were still rinsed in cell fractionation buffer and centrifuged. Cell disruption buffer was used for cell nuclei. U6 or GAPDH served as control for nuclear RNA or cytoplasm RNA.

### Chromatin immunoprecipitation (ChIP) assay

This assay was carried out with an EZ-Magna ChIP™ A/G Chromatin Immunoprecipitation Kit (Millipore) so as to explore the combination of FOXM1 and the LINC00152 promoter in line with the specification of manufacturer. RA FLSs were lysed and sonicated to be DNA fragments that were then precipitated with antibodies against IgG and FOXM1 (Abcam). After the immunoprecipitation, precipitated DNA was eluted and then underwent Quantitative Real-time PCR.

### Luciferase reporter assay

LINC00152 promoter was subcloned into the pGL3-basic vector (Genechem, Shanghai, China). Then, pGL3-LINC00152 promoter vectors and sh-FOXM1#1 or sh-FOXM1#2 or FOXM1 or their corresponding NC were co-transfected into RA FLSs cells. Simultaneously, wild-type or mutant sequences of miR-1270 in LINC00152 (LINC00152-WT or LINC00152-MUT; Genepharma) were subcloned into the pmirGLO luciferase reporter vector and then co-transfected into RA FLSs with miR-1270 mimics or its NC. After transfection via Lipotransfectamine 3000, the Dual-Luciferase Report Assay (Promega, Madison, WI, U.S.A.) was experimented.

### RNA pull-down assay

RA FLS were treated with biotin-labeled miR-1270-WT and biotin-labeled miR-204-MUT. At 48 h post-transfection, PBS washed the collected and specific lysate buffer (Ambion, Austin, Texas) incubated RA FLS for 10 min. M-280 streptavidin beads (Sigma-Aldrich St. Louis, MO) was applied for lysates incubation at 4°C for 3 h, of which beads were pre-coated with yeast tRNA and RNase-free BSA (Sigma-Aldrich St. Louis, MO). Trizol was for the combined RNA purification, following which LINC00152 and FOXM1 were measured by RT-qPCR.

### RNA immunoprecipitation (RIP) assay

Following the manufacturer’s directions, EZ-Magna RIP kit (Millipore, Billerica, MA, U.S.A.) was included for RIP performance. We scraped off RA FLS at roughly 80–90% confluency. RIP buffer comprising magnetic beads coupled with anti-Ago2 or anti-IgG (Millipore) was utilized to incubate RA FLS after the incubation with complete RIP lysis buffer. After digestion of Proteinase K, then immunoprecipitated RNA was acquired. A NanoDrop (Thermo Scientific) determined RNA concentration and a bioanalyser (Agilent, Santa Clara, CA, U.S.A.) evaluated the RNA quality. Furthermore, the presence of LINC00152, miR-1270 and FOXM1 was subjected to qRT-PCR analysis.

### FISH assay

Ribo™ Fluorescent in Situ Hybridization Kit (Ribobio Company, China) here was utilized as per the manufacturers’ guide. LINC00152 probe was designed via Ribobio Company, followed by Cy3 fluorescent dye labeling. Fluorescent in situ hybridization kit (RiboBio) was used as suggested for RNA FISH assay. A confocal laser-scanning microscope (Leica, Germany) detected fluorescence.

### Immunofluorescence analysis

Immunofluoresence staining utilized antibodies against β-catenin (anti-β-catenin) and the secondary antibodies Alexa Fluor 488 and 594 (Life Technologies Corp.). FV-1200 laser scanning confocal microscope was adopted for microscopy detection.

### Statistical analysis

Data from at least three separate experiments were expressed as the mean ± standard. Student’s *t*-test or ANOVA for statistical analysis was performed by SPSS 23.0 (IBM, Chicago, IL, U.S.A.). *P* below 0.05 was considered as statistically meaningful.

## Results

### LINC00152 facilitates the cell growth of RA FLS

**Figure 1:**
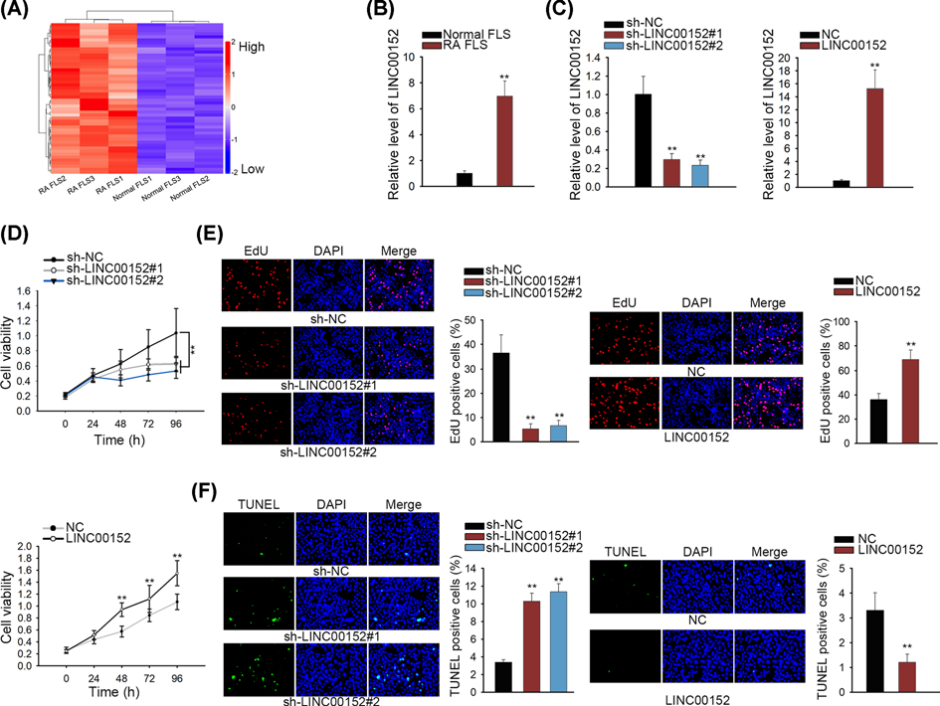
LINC00152 facilitates the growth of RA FLS (**A**) Top 50 up-regulated lncRNAs (fold change > 5.0) were selected out through microarray analysis. (**B**) LINC00152 expression in RA FLS and normal FLS were detected by qRT-PCR analysis. (**C**) qRT-PCR examination showed the overexpression or knockdown efficiency for LINC00152 in RA FLS. (**D** and **E**) CCK-8 and EdU assays were carried out to detect proliferation in RA FLS transfected with LINC00152-specific shRNAs or LINC00152 over expression vector. (**F**) Apoptosis in RA FLS was measured after silencing or overexpression of LINC00152; *P < 0.05, **P < 0.01.

At first, primary FLS was isolated from RA patients (RA FLS) or healthy patients (normal FLS). To screen out RA-associated lncRNAs, we applied a microarray analysis to find lncRNAs that were differentially expressed in RA FLS. As presented in Figure 1A, top 50 up-regulated lncRNAs were selected out due to their highest fold change (>5.0). Among which, LINC00152 presented the highest fold change. Up-regulation of LINC00152 in RA FLS was also proved by qRT-PCR (Figure 1B). To determine the correlation between LINC00152 expression and the biological processes of RA, gain- or loss-of function investigation was carried out in RA FLS. LINC00152 was silenced or overexpressed in RA FLS, and the transfection efficiency was measured and validated by qRT-PCR (Figure 1C). In CCK-8 and EdU assays, we disclosed that inhibition of LINC00152 expression weakened proliferative ability of RA FLS, whereas up-regulation of LINC00152 caused opposite results (Figure 1D,E). Cell cycle analysis supported that loss of LINC00152 augmented cells in G1/G0, and declined cells in S phase. Whereas augmentation of LINC00152 decreased cells in G1/G0, while promoted cells in S phase (Supplementary Figure S1A). Apoptosis was also assessed in indicated RA FLS. It was uncovered that silencing of LINC00152 induced cell apoptosis. However, overexpression of LINC00152 impeded apoptosis (Figure 1F). Taken together, LINC00152 promotes cell growth of RA FLS.

### LINC00152 activates Wnt/β-catenin signaling pathway and regulates nuclear translocation of β-catenin

**Figure 2:**
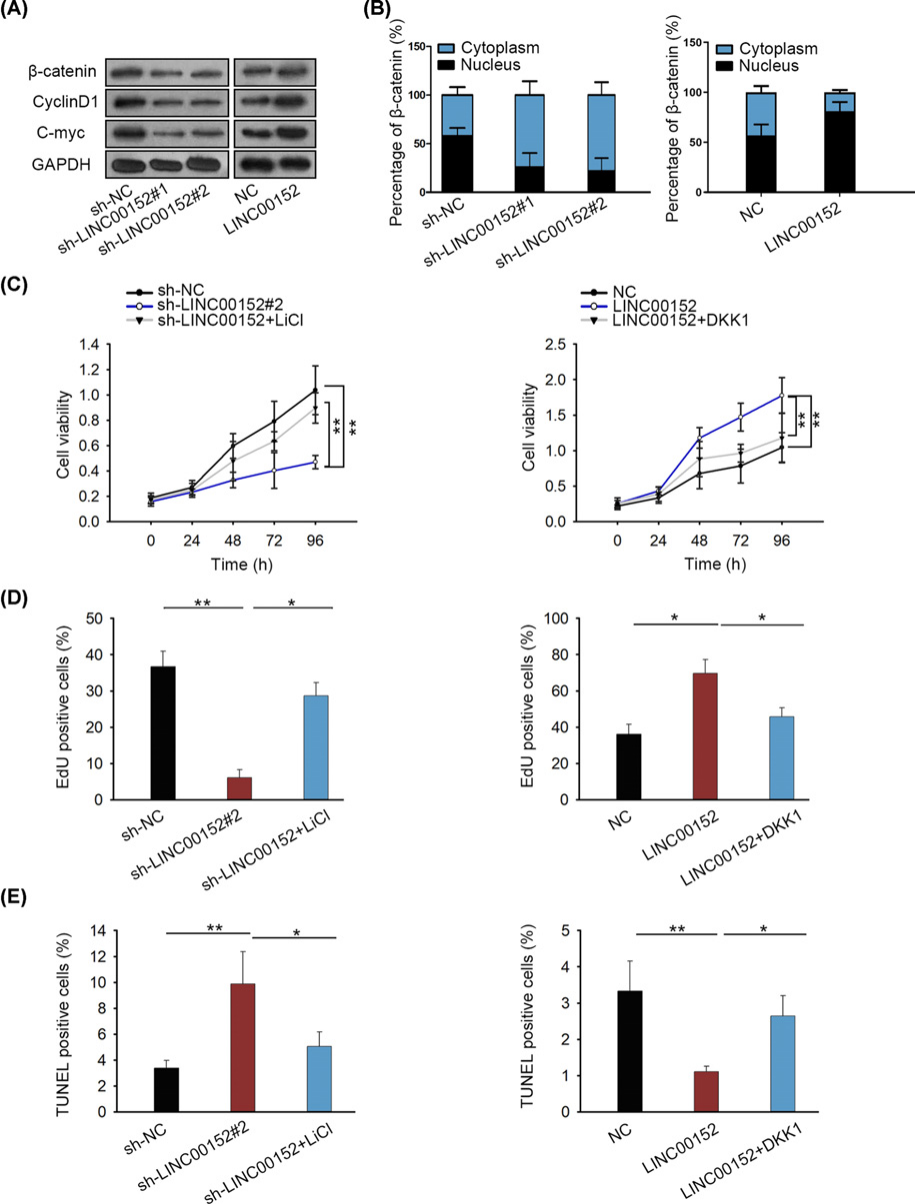
LINC00152 activates Wnt/β-catenin pathway and regulates nuclear translocation of β-catenin (**A**) The effect of LINC00152 on the Wnt/β-catenin pathway proteins (β-catenin, c-myc as well as cyclin D1) was assessed by Western blotting. (**B**) Nuclear translocation of β-catenin was detected in RA FLS after LINC00152 was overexpressed or silenced. (**C** and **D**) Proliferative ability RA FLS transfected with sh-LINC00152 or pcDNA-LINC00152 was measured after treatment with LiCl or DKK1 over expression vector. (**E**) Apoptosis in RA FLS was detected after treatment with indicated plasmids; *P < 0.05, **P < 0.01.

Wnt/β-catenin signaling can be mediators in biological processes of RA [16,17]. In previous studies, LINC00152 has been reported as a modulator of Wnt/β-catenin signaling pathway [18,19]. Here, we investigated the regulatory effect of LINC00152 on the protein levels of β-catenin, c-myc as well as cyclin D1 (core factors of Wnt/β-catenin signaling pathway). The levels of all these three proteins were positively regulated by LINC00152 (Figure 2A). Furthermore, cytoplasmic/nuclear fractionation assay revealed that LINC00152 prompted β-catenin translocate into nucleus of RA FLS (Figure 2B). Subsequent immunofluorescence analysis indicated the declined nuclear transposition of β-catenin upon LINC00152 depletion, whereas increased LINC00152 accelerated β-catenin translocation into RA FLS nuclei (Supplementary Figure S1B). Through TOP/FOP flash luciferase reporter assays, we determined that the activity of Wnt/β-catenin signaling was positively regulated via LINC00152 (Supplementary Figure S1C). To prove the mediation of Wnt/β-catenin signaling pathway in LINC00152-regulated RA FLS growth, LiCl (activator of Wnt/β-catenin signaling pathway) and DKK1 (inhibitor of Wnt/β-catenin signaling pathway) were applied to conduct rescue assays. According to results of cell proliferation assay, treatment of LiCl recovered cell proliferation suppressed by silenced LINC00152, whereas overexpression of DKK1 rescued LINC00152-induced cell proliferation (Figure 2C,D). Further, apoptosis was also examined in transfected RA FLS. It was uncovered that sh-LINC00152#1-induced apoptosis was reduced after RA FLS was treated with LiCl. Whereas cell apoptosis suppressed by LINC00152 overexpression was increased by introducing DKK1 over expression vector (Figure 2E). Based on above data, we demonstrate that LINC00152 promotes growth of RA FLS via activating Wnt/β-catenin signaling pathway.

### FOXM1 transcriptionally activates LINC00152 and acts as a downstream target of LINC00152

**Figure 3:**
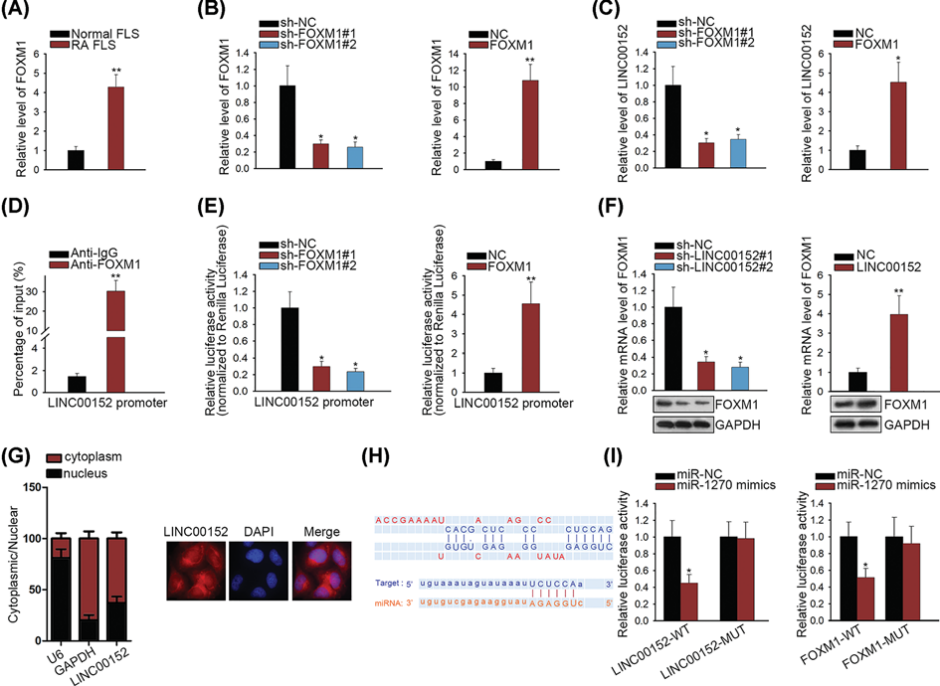
FOXM1 transcriptionally activates LINC00152 and acts as a downstream target of LINC00152 (**A**) qRT-PCR examination to detect FOXM1 expression in RA FLS and normal FLS. (**B**) Transfection efficiency for FOXM1 overexpression or silencing was identified through qRT-PCR. (**C**) LINC00152 expression was examined in RA FLS after overexpression or silencing of FOXM1. (**D**) ChIP assay was carried out in RA FLS to determine the affinity of FOXM1 to LINC00152 promoter. (**E**) The effect of FOXM1 on the luciferase activity of LINC00152 promoter was detected using luciferase reporter assay. (**F**) The effect of LINC00152 on FOXM1 mRNA or protein was detected by qRT-PCR and Western blotting. (**G**) Subcellular fractionation and FISH experiments to identify the cellular localization of LINC00152 in RA FLS. (**H**) Putative binding sequence of miR-1270 in LINC00152 and FOXM1 3′ UTR. (**I**) The interaction between miR-1270 and LINC00152 or FOXM1 was further verified by luciferase reporter assay; *P < 0.05, **P < 0.01.

Transcription activation is one of the main molecular mechanisms causing the activation of lncRNAs. With this regard, we designed mechanism experiments to find the upstream transcription activator of LINC00152. By screening from UCSC online database, FOXM1 is a potential transcription regulator for LINC00152. Based on previous reports, FOXM1 can act as either a transcription activator for lncRNAs [20–22] or an activator for Wnt/β-catenin signaling pathway [23–25]. In consistent with LINC00152, FOXM1 was also expressed at high level in RA FLS than that in normal FLS (Figure 3A). To analyze the regulatory impact of FOXM1 on LINC00152, FOXM1 was separately silenced and overexpressed in RA FLS, respectively (Figure 3B). As expected, silencing of FOXM1 led to the down-regulation of LINC00152, whereas overexpression of FOXM1 induced up-regulation of LINC00152 (Figure 3C). Furthermore, ChIP assay determined the affinity of FOXM1 to LINC00152 promoter in RA FLS (Figure 3D). The effect of FOXM1 on the luciferase activity of LINC00152 promoter was detected. Unsurprisingly, FOXM1 could enhance the luciferase activity of LINC00152 promoter (Figure 3E). Subsequently, we detected the regulation of LINC00152 on FOXM1 mRNA or protein. Intriguingly, both mRNA and protein levels of FOXM1 were positively regulated by LINC00152 (Figure 3F). To determine the modulatory pattern of LINC00152 in RA FLS, we applied subcellular fractionation assay and FISH to identify the cellular localization. Cytoplasmic localization of LINC00152 indicated that LINC00152 post-transcriptionally regulated FOXM1 in RA FLS (Figure 3G). Then, we investigated whether LINC00152 and FOXM1 involve in a ceRNA pathway. Through bioinformatics prediction, we uncovered that miR-1270 had complementary base paring with both LINC00152 and FOXM1 (Figure 3H). The interaction between miR-1270 and LINC00152 or FOXM1 was further verified by luciferase reporter assay (Figure 3I). Furthermore, RNA pull-down and RIP assays together proved co-expressed LINC00152, miR-1270 and FOXM1 in RISC (Supplementary Figure S1D,E). FISH assay disclosed co-localized LINC00152, miR-1270 and FOXM1. LINC00152 silencing led to miR-1270 promotion and FOXM1 reduction (Supplementary Figure S1F). Similarly, the positive effect of FOXM1 on Wnt/β-catenin signaling activity was confirmed by TOP/FOP flash luciferase reporter assay (Supplementary Figure S1G). Thus, we summarize that LINC00152 and FOXM1 form a positive feedback loop in RA FLS.

### FOXM1 promotes the growth of RA FLS via Wnt/β-catenin signaling pathway

**Figure 4:**
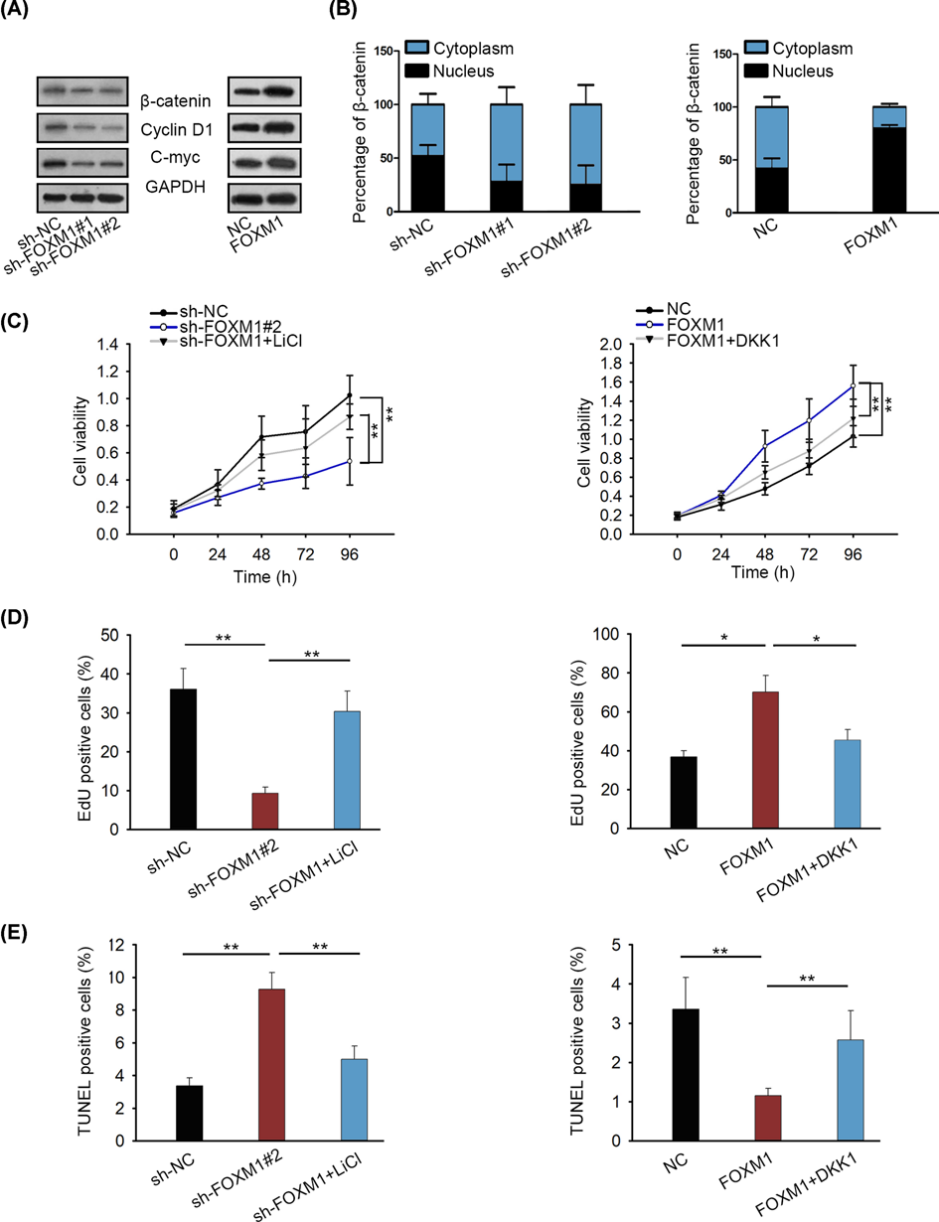
FOXM1 promotes the growth of RA FLS via Wnt/β-catenin pathway (**A**) Protein levels of Wnt/β-catenin pathway downstream genes in response to FOXM1 knockdown or overexpression were examined in RA FLS. (**B**) The influence of FOXM1 on β-catenin translocation in RA FLS. (**C** and **D**) Proliferative ability RA FLS transfected with sh-FOXM1 or pcDNA-FOXM1 was measured after treatment with LiCl or pcDNA-DKK1. (**E**) Apoptosis in indicated RA FLS was detected via TUNEL staining; *P < 0.05, **P < 0.01.

We further detected whether FOXM1 can act as a regulator for Wnt/β-catenin signaling pathway in RA FLS. The protein levels of β-catenin, c-myc as well as cyclin D1 were positively regulated by FOXM1 in RA FLS (Figure 4A). Furthermore, FOXM1 was proved to prompt β-catenin translocation into nucleus of RA FLS (Figure 4B). Similar results were also displayed in immunofluorescence, which illuminated the more nuclear β-catenin upon FOXM1 up-regulation and less nuclear β-catenin upon FOXM1 down-regulation (Supplementary Figure S1F). For functional assays, LiCl and DKK1 were applied to treat RA FLA transfected with sh-FOXM1#1 or FOXM1 expression vector. According to results of cell proliferation assay, treatment of LiCl recovered cell proliferation suppressed by sh-FOXM1#1, whereas overexpression of DKK1 attenuated the effect of FOXM1 overexpression on proliferation (Figure 4C,D). Further, TUNEL assay revealed that sh-FOXM1#1-induced apoptosis was partly reversed after treatment with LiCl. Whereas cell apoptosis suppressed by FOXM1 overexpression was increased by introducing DKK1 over expression vector (Figure 4E). These data suggest that FOXM1 boosts RA FLS growth via activation of Wnt/β-catenin pathway.

## Discussion

Experimental results of this work are correlated with RA progression. Cartilage destruction, synovial hyperplasia and joint dysfunction are main pathogenetic characteristics of RA. Unclear pathogenesis is a challenge to the treatment of RA. Although the molecular mechanisms involved in pathogenesis of RA remain largely unknown, epigenetic modification and post-transcriptional regulation have been identified to participate in initiation and progression of RA [26,27]. Current evidence has suggested that lncRNAs are essential mediators in various biological activities [28]. Although lncRNAs have been widely reported in various human diseases, the mechanism and function of LINC00152 in RA progression remain to be explored. The main finding of the present study is that LINC00152 is a facilitator in RA progression. And LINC00152 induced proliferation and suppressed apoptosis in RA FLS. This finding will contribute to find novel diagnostic biomarker in RA.

Wnt/β-catenin signaling pathway is acknowledged as a crucial participant in various diseases, including RA Wnt/β-catenin signaling pathway being mediators in RA [16,17]. More importantly, LINC00152 has been proved to be a regulator for Wnt/β-catenin signaling pathway in human cancers [18,19]. In this regard, we investigated whether there is regulatory mechanism between LINC00152 and Wnt/β-catenin signaling. Our data suggested that LINC00152 enhanced Wnt/β-catenin signaling activity and promoted the nuclear transcription of β-catenin in RA FLS. Further functional experiments prompted us to conclude that Wnt/β-catenin signaling pathway was implicated in LINC00152-modulated RA FLS growth.

Upon mechanism investigation, we unveiled that FOXM1 could transcriptionally activate LINC00152 in RA FLS. Furthermore, FOXM1 has been reported to function in ceRNA by cooperating with lncRNA–miRNA axis [29,30]. In the present study, we identified cytoplasmic localization of LINC00152 in RA FLS. Further mechanism study revealed that LINC00152 and FOXM1 could competitively bind with miR-1270 in RA FLS.

As we mentioned above, FOXM1 might be a mediator for Wnt/β-catenin signaling pathway. Thus, we conducted experiments to validate the above speculation in RA FLS. In consistent with LINC00152, FOXM1 could activate Wnt/β-catenin signaling and prompted β-catenin to translocate into nucleus. Through rescue assays, we demonstrated that FOXM1 promoted growth of RA FLS via Wnt/β-catenin signaling pathway. All in all, the present study revealed that FOXM1/LINC00152 positive feedback loop promoted RA FLS growth via Wnt/β-catenin pathway. In present work, we uncovered the function and mechanism of a novel molecular mechanism in RA FLS growth. Our findings may contribute to providing new promising diagnostic biomarker or molecule-directed treatment for RA. To deal with the disadvantages in this manuscript, we will investigate the clinical significance of FOXM1/LINC00152 axis in our future study with clinical samples. Besides, the specific downstream functional genes of WNT pathway will be further interrogated in contributing to RA pathogenicity.

## Supplementary Material

Supplementary Figure S1

## Abbreviations

ANOVA: Analysis of variance
BCA: Bicinchoninic acid
CCK-8: Cell Counting Kit-8
ChIP: chromatin immunoprecipitation
DMEM: Dulbecco’s modified Eagle’s medium
EdU: 5-ethynyl-2′-deoxyuridine
FBS: Fetal bovine serum
FISH: Fluorescence in Situ Hybridization
FLS: Fibroblast-like synoviocytes
FOXM1: forkhead box M1
GAPDH: Glyceraldehyde-3-phosphate dehydrogenase
IgG: Immunoglobulin G
LINC00152: cytoskeleton regulator RNA (also known as CYTOR)
LncRNA: long non-coding RNA
NC: negative control
PVDF: Polyvinylidene fluoride
qRT-PCT: Quantitative Real-time PCR
RA: rheumatoid arthritis
RIP: RNA Binding Protein Immunoprecipitation
RISC: RNA-induced silencing complex
SDS-PAGE: Sodium dodecyl sulfate-polyacrylamide gel electrophoresis
shRNA: Short hairpin RNA
TUNEL: Terminal deoxynucleotidyl transferase dUTP nick end labeling
UTR: Untranslated region

## References

[1] Churov A.V., Oleinik E.K. and Knip M. (2015) MicroRNAs in rheumatoid arthritis: altered expression and diagnostic potential. Autoimmun. Rev. 14, 1029–1037 10.1016/j.autrev.2015.07.005 26164649

[2] Firestein G.S. (2003) Evolving concepts of rheumatoid arthritis. Nature 423, 356–361 10.1038/nature01661 12748655

[3] Rossini M., Rossi E., Bernardi D., Viapiana O., Gatti D., Idolazzi L. et al. (2014) Prevalence and incidence of rheumatoid arthritis in Italy. Rheumatol. Int. 34, 659–664 10.1007/s00296-014-2974-6 24610538

[4] Ceribelli A., Nahid M.A., Satoh M. and Chan E.K. (2011) MicroRNAs in rheumatoid arthritis. FEBS Lett. 585, 3667–3674 10.1016/j.febslet.2011.05.020 21600203 PMC3168677

[5] van der Linden M.P., le Cessie S., Raza K., van der Woude D., Knevel R., Huizinga T.W. et al. (2010) Long-term impact of delay in assessment of patients with early arthritis. Arthritis Rheum. 62, 3537–3546 10.1002/art.27692 20722031

[6] Ji H., Hui B., Wang J., Zhu Y., Tang L., Peng P. et al. (2019) Long noncoding RNA MAPKAPK5-AS1 promotes colorectal cancer proliferation by partly silencing p21 expression. Cancer Sci. 110, 72–85 10.1111/cas.13838 30343528 PMC6317943

[7] Zhu L., Yang N., Li C., Liu G., Pan W. and Li X. (2018) Long noncoding RNA NEAT1 promotes cell proliferation, migration, and invasion in hepatocellular carcinoma through interacting with miR-384. J. Cell. Biochem. 120, 1997–2006 10.1002/jcb.27499 PMC6587825 30346062

[8] Imamura K. and Akimitsu N. (2014) Long Non-Coding RNAs Involved in Immune Responses. Front. Immunol. 5, 573 10.3389/fimmu.2014.00573 25431574 PMC4230175

[9] Wu G.C., Pan H.F., Leng R.X., Wang D.G., Li X.P., Li X.M. et al. (2015) Emerging role of long noncoding RNAs in autoimmune diseases. Autoimmun. Rev. 14, 798–805 10.1016/j.autrev.2015.05.004 25989481

[10] Kong J.S., Yoo S.A., Kim H.S., Kim H.A., Yea K., Ryu S.H. et al. (2010) Inhibition of synovial hyperplasia, rheumatoid T cell activation, and experimental arthritis in mice by sulforaphane, a naturally occurring isothiocyanate. Arthritis Rheum. 62, 159–170 10.1002/art.25017 20039434

[11] Izquierdo E., Canete J.D., Celis R., Del Rey M.J., Usategui A., Marsal S. et al. (2011) Synovial fibroblast hyperplasia in rheumatoid arthritis: clinicopathologic correlations and partial reversal by anti-tumor necrosis factor therapy. Arthritis Rheum. 63, 2575–2583 10.1002/art.30433 21547893

[12] Buckland J. (2014) Rheumatoid arthritis: TNF targets histones to loosen chromatin in RA FLS. Nat. Rev. Rheumatol. 10, 636 10.1038/nrrheum.2014.165 25247409

[13] Wang G., Tang L., Zhang X. and Li Y. (2019) LncRNA DILC participates in rheumatoid arthritis by inducing apoptosis of fibroblast-like synoviocytes and down-regulating IL-6. 39, BSR20182374 10.1042/BSR20182374 PMC6499449 30944206

[14] Ye Y., Gao X. and Yang N. (2018) LncRNA ZFAS1 promotes cell migration and invasion of fibroblast-like synoviocytes by suppression of miR-27a in rheumatoid arthritis. Hum. Cell 31, 14–21 10.1007/s13577-017-0179-5 28721682

[15] Mo B.Y., Guo X.H., Yang M.R., Liu F., Bi X., Liu Y. et al. (2018) Long Non-Coding RNA GAPLINC Promotes Tumor-Like Biologic Behaviors of Fibroblast-Like Synoviocytes as MicroRNA Sponging in Rheumatoid Arthritis Patients. Front. Immunol. 9, 702 10.3389/fimmu.2018.00702 29692777 PMC5902673

[16] Li G.Q., Fang Y.X., Liu Y., Meng F.R., Wu X., Zhang C.W. et al. (2019) MALAT1-driven inhibition of Wnt signal impedes proliferation and inflammation in fibroblast-like synoviocytes through CTNNB1 promoter methylation in rheumatoid arthritis. Hum. Gene Ther. 30, 1008–1022 10.1089/hum.2018.212 30909750

[17] Wu J., Fan W., Ma L. and Geng X. (2018) miR-708-5p promotes fibroblast-like synoviocytes’ cell apoptosis and ameliorates rheumatoid arthritis by the inhibition of Wnt3a/beta-catenin pathway. Drug Design Dev. Ther. 12, 3439–3447 10.2147/DDDT.S177128 30349197 PMC6186895

[18] Xian-Li T., Hong L., Hong Z., Yuan L., Jun-Yong D., Peng X. et al. (2019) Higher Expression of Linc00152 Promotes Bladder Cancer Proliferation and Metastasis by Activating the Wnt/beta-Catenin Signaling Pathway. Med. Sci. Monit. 25, 3221–3230 10.12659/MSM.913944 31042695 PMC6507494

[19] Shan Y., Ying R., Jia Z., Kong W., Wu Y., Zheng S. et al. (2017) LINC00052 Promotes Gastric Cancer Cell Proliferation and Metastasis via Activating the Wnt/beta-Catenin Signaling Pathway. Oncol. Res. 25, 1589–1599 10.3727/096504017X14897896412027 28337962 PMC7841087

[20] Chen F., Bai G., Li Y., Feng Y. and Wang L. (2017) A positive feedback loop of long noncoding RNA CCAT2 and FOXM1 promotes hepatocellular carcinoma growth. Am. J. of Cancer Res. 7, 1423–1434 28744394 PMC5523025

[21] Li Y. and Zhang T. (2018) Targeting the FOXM1-regulated long noncoding RNA TUG1 in osteosarcoma. 109, 3093–3104 10.1111/cas.13765 PMC6172046 30099814

[22] Xu M.D., Wang Y., Weng W., Wei P., Qi P., Zhang Q. et al. (2017) A Positive Feedback Loop of lncRNA-PVT1 and FOXM1 Facilitates Gastric Cancer Growth and Invasion. Clin. Cancer Res. 23, 2071–2080 10.1158/1078-0432.CCR-16-0742 27756785

[23] Quan M., Cui J., Xia T., Jia Z., Xie D., Wei D. et al. (2015) Merlin/NF2 Suppresses Pancreatic Tumor Growth and Metastasis by Attenuating the FOXM1-Mediated Wnt/beta-Catenin Signaling. Cancer Res. 75, 4778–4789 10.1158/0008-5472.CAN-14-1952 26483206 PMC4651817

[24] Gong A. and Huang S. (2012) FoxM1 and Wnt/beta-catenin signaling in glioma stem cells. Cancer Res. 72, 5658–5662 10.1158/0008-5472.CAN-12-0953 23139209 PMC3500394

[25] Zhang N., Wei P., Gong A., Chiu W.T., Lee H.T., Colman H. et al. (2011) FoxM1 promotes beta-catenin nuclear localization and controls Wnt target-gene expression and glioma tumorigenesis. Cancer Cell 20, 427–442 10.1016/j.ccr.2011.08.016 22014570 PMC3199318

[26] Smolen J.S., Aletaha D. and McInnes I.B. (2016) Rheumatoid arthritis. Lancet 388, 2023–2038 10.1016/S0140-6736(16)30173-8 27156434

[27] Malmstrom V., Catrina A.I. and Klareskog L. (2017) The immunopathogenesis of seropositive rheumatoid arthritis: from triggering to targeting. Nat. Rev. Immunol. 17, 60–75 10.1038/nri.2016.124 27916980

[28] Li R., Zhu H. and Luo Y. (2016) Understanding the Functions of Long Non-Coding RNAs through Their Higher-Order Structures. Int. J. Mol. Sci. 17, 702 10.3390/ijms17050702 PMC4881525 27196897

[29] Li C.F., Li Y.C., Wang Y. and Sun L.B. (2018) The Effect of LncRNA H19/miR-194-5p Axis on the Epithelial-Mesenchymal Transition of Colorectal Adenocarcinoma. Cell. Physiol. Biochem. 50, 196–213 10.1159/000493968 30278464

[30] Wei Y., Sun Q., Zhao L., Wu J., Chen X., Wang Y. et al. (2016) LncRNA UCA1-miR-507-FOXM1 axis is involved in cell proliferation, invasion and G0/G1 cell cycle arrest in melanoma. Med. Oncol. 33, 88 10.1007/s12032-016-0804-2 27389544

