# Supplementary material for: FOXM1/LINC00152 feedback loop regulates proliferation and apoptosis in rheumatoid arthritis fibroblast-like synoviocytes via Wnt/β-catenin signaling pathway
Source: Biosci Rep. 2020 Jan 21;40(1):BSR20191900. doi: 10.1042/BSR20191900 (PMC6974425; doi:10.1042/BSR20191900)
Supplement: Supplementary Figure S1 [file BSR-2019-1900_supp.pdf]

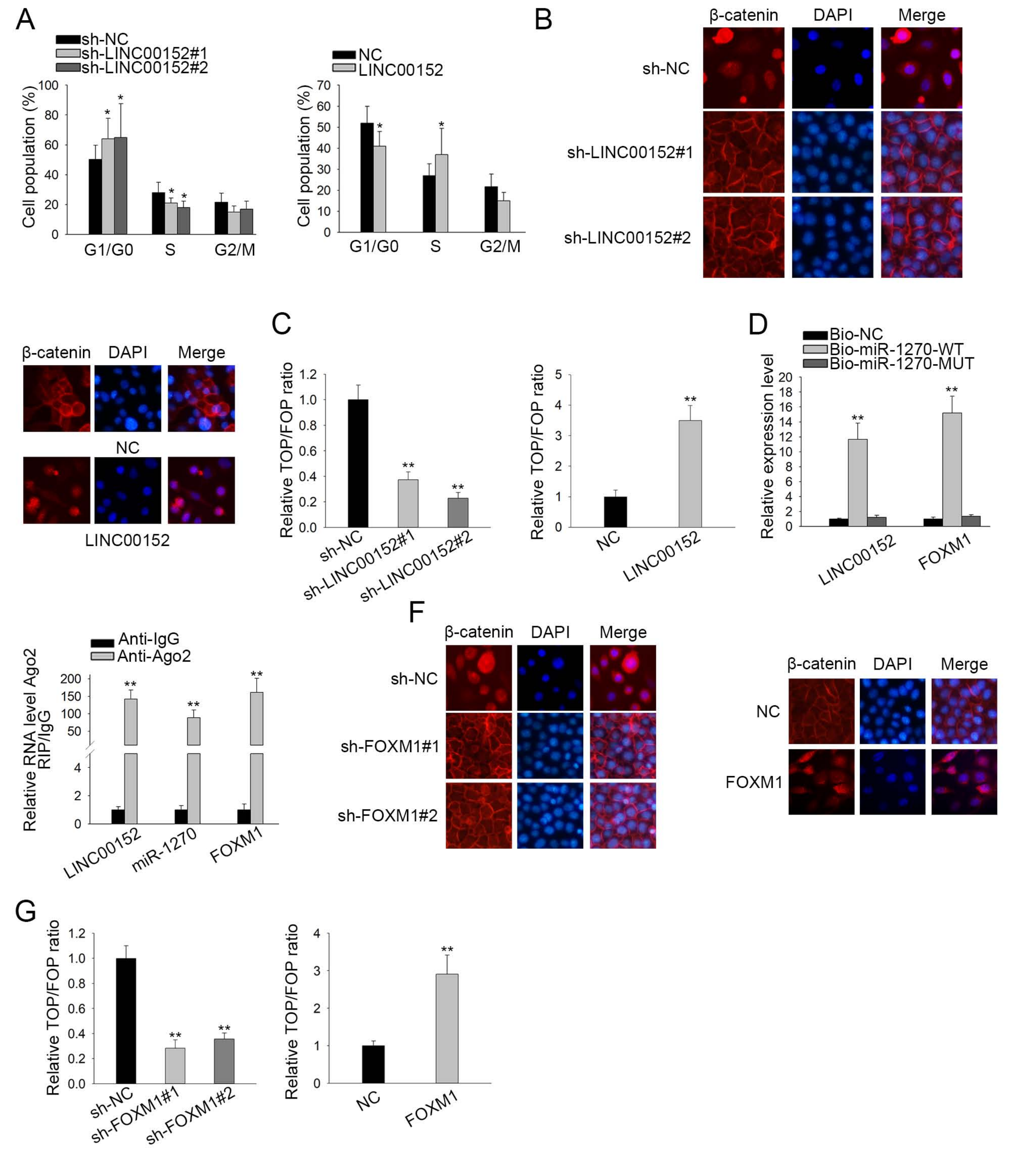
A
sh-NC
sh-LINC00152#1
sh-LINC00152#2
Cell population (%)
G1/G0
S
G2/M
NC
LINC00152
B
β-catenin
DAPI
Merge
sh-NC
sh-LINC00152#1
sh-LINC00152#2
NC
LINC00152
C
Relative TOP/FOP ratio
D
Bio-NC
Bio-miR-1270-WT
Bio-miR-1270-MUT
Relative expression level
FOXM1
Anti-IgG
Anti-Ago2
Relative RNA level Ago2 RIP/IgG
miR-1270
F
sh-FOXM1#1
sh-FOXM1#2
G

Figure S1 (A) RA FLS cell cycle was analyzed via flow cytometry following LINC00152 augmentation or inhibition. (B) Immunofluorescence assay illuminated the impacts of LINC00152 on β-catenin nuclear translocation. (C) The usage of TOP/FOP flash luciferase reporter assay for the impact of LINC00152 on Wnt/β-catenin signaling pathwayactivity. (D) RNA pull-down analysis of interaction among LINC00152, miR-1270 and FOXM1. (E) RIP assay probed the interplay among LINC00152, miR-1270 and FOXM1. (F) Immunofluorescence assay investigated the effects of FOXM1 on β-catenin nuclear accumulation. (G)The appliance of TOP/FOP flash luciferase reporter assay to determine the influence of FOXM1on Wnt/β-catenin signaling pathwayactivity.$^{*}P < 0.05$, $^{**}P < 0.01$.
